# Pharmacokinetics and pharmacodynamics in the treatment of cutaneous leishmaniasis – challenges and opportunities

**DOI:** 10.1039/d0md00343c

**Published:** 2021-01-07

**Authors:** Katrien Van Bocxlaer, Simon L. Croft

**Affiliations:** Department of Biology, York Biomedical Research Institute, University of York York YO10 5DD UK Katrien.vanbocxlaer@york.ac.uk +44 (0) 19 0432 8855; Department of Infection Biology, London School of Hygiene & Tropical Medicine London WC1E 7HT UK

## Abstract

Pharmacological efficacy is obtained when adequate concentrations of a potent drug reach the target site. In cutaneous leishmaniasis, a heterogeneous disease characterised by a variety of skin manifestations from simple nodules, skin discoloration, plaques to extensive disseminated forms, the parasites are found in the dermal layers of the skin. Treatment thus involves the release of the active compound from the formulation (administered either topically or systemically), it's permeation into the skin, accumulation by the local macrophages and further transport into the phagolysosome of the macrophage. The pharmacodynamic activity of a drug against the parasite is relatively straight forward to evaluate both *in vivo* and *in vitro*. The pharmacokinetic processes taking place inside the skin are more complex to elucidate due to the multi-lamellar structure of the skin, heterogeneous distribution of drugs within the tissue, the difficulty of accessing the site of infection complicating sampling and the lack of surrogate markers reflecting the activity of a drug in the skin. This review will discuss the difficulties encountered when investigating drug distribution, PK PD relationships and efficacy in the skin with a focus on cutaneous leishmaniasis treatment.

## Abstract 2

Important pharmacokinetic and -dynamic parameters for the drug discovery and development of new treatments for cutaneous leishmaniasis.

## Graphical abstract

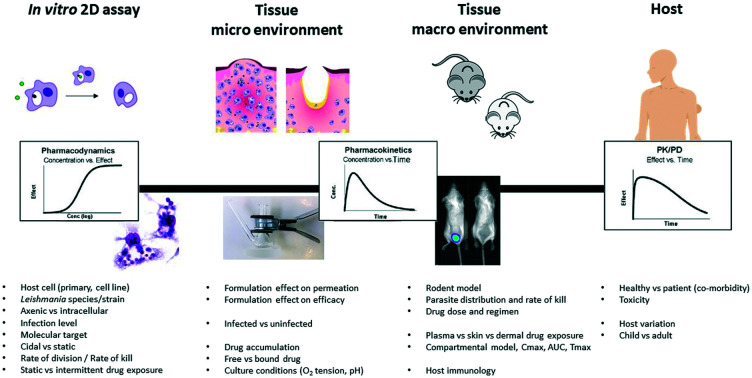

## Introduction

Cutaneous leishmaniasis (CL) is a poverty-related neglected tropical skin disease that manifests as a variety of cutaneous symptoms from distinct simple nodules and ulcers to extended plaques and disseminated forms. Whilst localised lesions take 6 to 18 months to heal, a small percentage of cases progress to chronic infections. The visibility of the skin lesions re-enforce feelings of isolation, cause stigma and discrimination^1,2^ – this psychological and socio-economic impact places cutaneous diseases as 4th leading cause of non-fatal disease burden worldwide.^3–6^

Despite these findings, the incentive for drug discovery and development is limited in particular for neglected tropical skin diseases and can in part be explained by: (i) the generally low benefit – risk as skin diseases rarely cause fatalities, (ii) an inadequate insight in the pathophysiology of skin diseases complicating the delivery and testing of novel compounds, (iii) the lack of translation between *in vitro* drug assays (enzymatic and cellular) and *in vivo* models and, (iv) the limited tools and surrogate markers to measure pharmacodynamic and pharmacokinetic parameters such as drug distribution in the skin.^7,8^

The chemotherapeutic arsenal for all forms of CL is unsatisfactory and would benefit from the addition of a local topical treatment for small lesions limited in number and systemic oral formulation with or without an immunomodulator for more complex forms of CL.^9^ With a well-populated pipeline for visceral leishmaniasis (VL), there is currently a renewed focus on drug discovery for CL.^10^ However, although both diseases are caused by the same genus of parasite, the drug target product profiles differ substantially with a requirement for different PK profiles and compound formulations.^10^ This is important as lead compounds with optimal PK profiles have been directly related to overall clinical success^11,12^ and thus clinical investment.^13^

## The skin as the site for drug activity

**Fig. 1:**
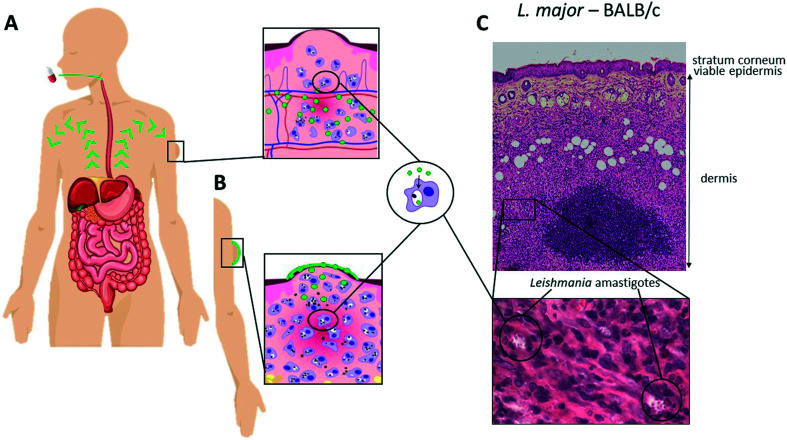
Schematic of the drug pathway for oral (A) *versus* topical (B) drug administration for cutaneous leishmaniasis. (C) Skin morphology in *L. major* infected BALB/c mouse skin (H&E stain, top panel ×80 magnification, bottom panel ×400 magnification).

The location of the parasites (Fig. 1(C)) in the skin offers the opportunity for localised or topical treatments (Fig. 1(B)) of single and uncomplicated lesions in addition to systemic treatment options (Fig. 1(A)). The former is attractive as it minimises systemic adverse events and drug interactions and is easy to administer, potentially enhancing patient compliance. However, there are five major challenges: (i) the deeper dermal infection, unlike many superficial fungal and bacterial infections, requires formulation design to ensure maximum drug release and retention (lowering the risk of drug elimination to the haemo-lymphatic sink); (ii) the formidable multi-lamellar skin barrier with different layers restricts passive drug permeation processes (partitioning and/or diffusion) depending on the physicochemical properties of the drug. Mathematical modelling of experimental data have provided a better understanding of the passive permeation processes and allowed the identification of key drug properties such as molecular size, solubility and partition coefficient (Table 1),^14,15^ (iii) the necessity of drug accumulation into macrophages in the dermal layer, which is not a property of all antimicrobials,^16^ but may benefit from the pH gradient leading to the acidic phagosomal vacuole (pH∼5) where the *Leishmania* amastigotes survive and multiply;^17^ (iv) the changes in skin and dermal structure as a consequence of the infection with resulting epidermal changes leading to water loss,^18^ and inflammation leading to changes in drug accumulation^18,19^ and effect of immunomodulators,^20^ and (v) the different immunopathologies caused by different species of *Leishmania* with different immune cell profiles, for example the implication of CD8^+^ T cells in the excessive tissue reaction during *L. braziliensis* infection.^20,21^ These factors impact upon drug activity and should be given more consideration in both drug and formulation design to ensure appropriate distribution and release; specific examples are discussed below.

**Table 1:** Current drugs used for CL: range and general physicochemical properties^18,31,110^

	Amphotericin B	Paromomycin sulfate	Miltefosine	Pentamidine diisethionate	Sodium stibogluconate	Meglumine antimoniate
Partition coefficient (AlogP)	−2.6	−8.7	3.7	4.0	−3.8	−4.2
Solubility in water (mg ml^−1^)	<0.001	>20	>2.5	>20	>1	>300
Molecular weight (g mol^−1^)	924	714	408	593	680	525
pKa	5.7 and 10	5.7 to 8.8	2	11.5 and 12.9	−3 to 2.3	9.1 to 12.7

Contrary to most antibacterial and antifungal products that target avascular superficial layers of the skin such as the stratum corneum and viable epidermis, antileishmanial drugs require transport to the deeper vascularised dermis, a process that involves both diffusion, through what is essentially considered an aqueous environment, and additional mechanisms such as convective blood, lymphatic and interstitial flow.^22–24^ Pharmacokinetic modelling of published human cutaneous deeper tissue microdialysis data suggests a more active role of convective transport for highly plasma protein bound drugs.^22,24^ In contrast, high protein binding might also contribute to increased elimination of the drug from the skin tissue to the lymphatic and blood capillary transport^25^ highlighting the need for a balance between the advantageous deeper tissue transport and prolonged skin retention whilst minimising elimination in order to ensure drug efficacy in the dermis.

Systemic drug delivery, which ensures broad distribution of a drug into the skin is especially advisable for the more severe and complex forms of CL at risk of dissemination, mucosal involvement, recurring lesions or unresponsiveness to treatment. The skin, as most peripheral tissues, hosts either continuous and/or fenestrated blood vessels that serve as a semipermeable barrier between the blood and the tissue. Two barriers limit transport from the microcapillaries into the skin (i) a “charge barrier” or glycocalyx layer on the endothelial cells^26,27^ and, (ii) a “size barrier” that mainly relies on interendothelial junctions between endothelial cells.^28,29^ With the latter restricting paracellular transport, drug transport mainly occurs across the endothelial cells facilitating diffusion of small lipophilic compounds.^30^ Several intrinsic and extrinsic factors may influence passive diffusion across endothelial cells including blood flow – tissue mass ratio also perfusion rate, the extent of plasma protein and tissue binding, regional pH differences and capillary permeability.

The impact of the route of administration for skin drug delivery is apparent from studies showing the lack of activity of DNDI-0690, a nitroimidazole currently undergoing clinical trials as treatment for VL,^31^ against experimental CL when applied topically as a saturated solution providing maximal flux. When administered orally, DNDI-0690 was able to effectively clear all parasites from the skin.^32^ Another example was observed when evaluating the efficacy of benzoxaborole compounds LSH001 and LSH003 in the same model; topical application of LSH003 was unable to reduce the lesion size progression (and parasite load) whereas oral administration of the drug reduced this load by 50%. The opposite effect was seen for LSH001, which halted lesion size progression upon topical administration whilst lacking activity upon oral administration.^33^

The selective barriers to drug delivery related to the administration route are also exemplified for paromomycin and amphotericin B. Whereas intramuscular injections of paromomycin may lead to parasite clearance, topical application either requires (i) pore forming excipients to stimulate drug transport across the stratum corneum^34^ or (ii) debridement of the lesion and application of the formulation directly onto the wound bed followed by occlusion as was performed in clinical trials of WR279,396 with variable success.^35^ Similarly, many experimental formulations containing amphotericin B were tested with limited results. Anfoleish, an oil-in-water cream containing 3% amphotericin B was tested in CL patients who presented with open ulcers. The cream was applied twice or thrice daily and whereas safety was evident, the limited cure rate (∼30%) did not support further clinical development.^36^ Given the high potency of amphotericin B (submicromolar range) against *Leishmania* species, the observed inefficacy of Anfoleish (and other topical formulations) is most likely due to an inability of this polyene antibiotic to permeate further into the skin to reach the parasites. This might be a result of its high molecular weight in addition to its tendency to form oligomers resulting in a large hydrophobic surface area and a loss of selectivity for ergosterol-rich membranes.^37^ The liposomal formulation (AmBisome®) when administered by intraveneous infusion, however, has been used to treat CL with successful results.^38,39^ Yet another example of a molecule with non-ideal skin permeability is miltefosine. Even though initially marketed as a topical treatment for skin metastasis of breast cancer, this and other experimental formulations of miltefosine never led to further topical product development for CL even though it has efficacy against multiple cutaneous forms of leishmaniasis upon oral administration.^40–42^

The above examples should therefore stimulate a focus of efforts on new chemical entities with more appropriate physicochemical properties for skin drug distribution rather than reformulating known antileishmanial compounds lacking adequate skin permeability.

## Drug development paradigm

### Pharmacodynamics

The PD parameter for antibacterial and antifungal compounds has frequently been based upon the MIC values,^43,44^ which are also the basis for standardization of antimicrobial drugs.^45,46^ In studies on *Leishmania* species MIC values are not used rather the standard has been EC_50_/EC_90_ values normally derived from the dose response curve of a 48/72 hour *Leishmania* amastigote/macrophage assay. These assays (described extensively elsewhere^47^) have been used either for high throughput screening (with macrophage-like cell lines^48^) or to identify species susceptibility differences (with mouse peritoneal macrophages^49,50^). The intracellular amastigote macrophage derived values (EC_50_ preferred for efficacy/relative potency whereas EC_90_ preferred for PK/PD analyses) have the advantages of: (i) using the relevant amastigote form of the parasite, (ii) being reproducible with many decades of data for standard drugs, (iii) being able to show the key PD differences in susceptibility between strains/species, (iv) being suitable for drug resistance studies, and (v) being adaptable for rate-of-kill studies. As such they are included in the classical PK–PD figures including calculations of exposure.

However, this is an *in vitro* parameter, and like in all antimicrobial studies^51^ it has several critical limitations: (i) the set time period and concentration of drug exposure, which is critically different from the transient nature of the *in vivo* PK exposure in animal models and humans, (ii) the different properties of host cells subject to differences in nutrient supply, O_2_ tensions and flow (see ref. 17 for further discussion on impact on *Leishmania* and^52^ for effect of flow on drug activity) and (iii) does not take into account the *in vivo* survival and division of pathogens which may have fast dividing as well as quiescent populations.^53,54^ Hence the determination of the *in vivo* PD parameter, must determine skin amastigote burden over a dose range using qPCR or other methods. This will enable relevant the ED_50/90_ values to be determined. When these values are linked to exposure measured by concentration of the drug in skin, there is the essential data for analysis of PK/PD relationships. For *in vivo* analysis of rate-of-kill currently only serial sacrifice of treated animals is used. These approaches address concerns about *in vitro* derived PD values linked to the promised ‘predictivity’ of the PK PD model.

### PK PD relationships in the skin

**Fig. 2:**
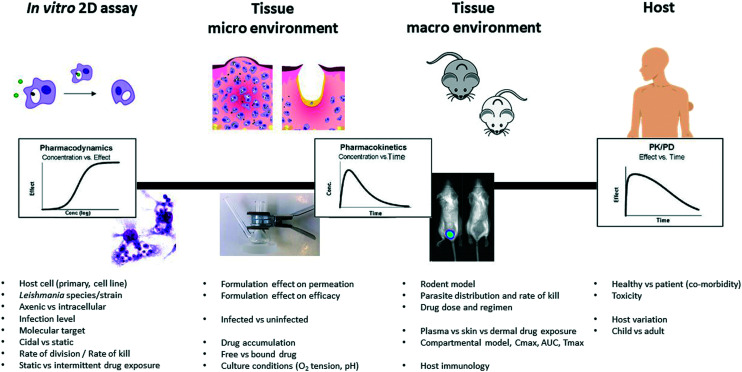
Diverse experimental tools and their respective PK and PD parameters (adapted from,^17^ with permission from parasitology, copyright 2017).

Over the past two decades it has become increasingly important in antimicrobial research to determine the relationship between the PK and PD of both novel compounds during lead optimisation, to support selection of compounds that achieve appropriate exposure in infected tissues, as well as for the selection of dose regimes for clinical trials (Fig. 2). Antimicrobials are generally divided into those that: (i) exhibit concentration-dependent killing (for example, aminoglycosides, fluoroquinolones), for which the area under the concentration-time curve (AUC) and peak concentration in relation to the MIC or EC_90_ of the pathogen, that is the AUC/MIC and Cmax/MIC, are the main PK/PD drivers that correlate with efficacy, and (ii) exhibit time-dependent killing (for example, beta-lactams and macrolides), where the time that the drug concentration exceeds the MIC (% *T* > MIC) is the major parameter determining efficacy. Dose-fractionation studies, in which the same total drug exposure is administered using different dosing intervals, are often used to indicate the key parameters in this analysis.

Only in the past decade has the PK/PD relationship of antileishmanial drugs for CL been investigated, led by Dorlo who with miltefosine established that the elimination from the body is best described by a two-compartment disposition model with an initial and terminal elimination phase (half-lives of 7 and 31 days, respectively). The latter inevitably led to prolonged sub therapeutic drug exposure with clinical implications such as parasite resistance development and toxicity related events.^55^ More recently, differences in plasma and intracellular miltefosine concentrations between children and adults were registered – key PK parameters such as area under the concentration-time curve and maximum concentration were found significantly lower for children. With no differences in strain susceptibilities, these outcomes urged for adapted dose regimens for children infected with new world CL^56,57^ and VL.^58^ Similarly, the distribution of antimony in the blood and intracellular peripheral blood mononuclear cell (PBMC) was measured upon treatment with meglumine antimoniate in patients infected with *L. Viannia* species and revealed a delayed and reduced antimony permeation into the PBMCs compared to the blood.^59^ Average measured PBMC concentrations ranged around 6.6 ng Sb/mL which is far below the reported *in vitro* EC_50_ values against *L. Viannia* strains (1–15 μg Sb/mL).^60^ Regardless of the rapid and complete drug distribution of antimony from the blood to healthy and diseased skin,^61^ this study demonstrates the unfavourable PK/PD profile of antimonial drugs. The importance of such studies for the clinic is clear. However, more work is required to investigate skin drug exposure in relation to blood distribution for all the different CL forms.

A major concern is how to translate the PK/PD profile from animal models to effective treatment regimens in humans and to ensure that: (i) both plasma and site of infections PK (and PD where possible) parameters are determined, including (ii) differences between these parameters in infected and uninfected animals.^17^ The impact of pathology on pharmacology might seem obvious for systemic drug delivery that relies on adequate perfusion of the target tissue which might be affected by the destruction of blood and lymphatic capillaries in necrosis and ulcer formation. Pathology is also associated with hypoxia which if it persists can dramatically impair tissue healing including the synthesis of collagen, the predominant element of the connective tissue.^62,63^ More importantly, it can interfere with the NOS_2_-dependent leishmanicidal activity of macrophages.^64^ With oxygen levels falling as low as 2.8% in lesions reaching their maximum dimensions,^65^ oxygenation is approximately half of the 5% generally used in standard drug susceptibility assays. Hence, local tissue oxygenation may contribute to the persistence of *Leishmania*.^66^ Furthermore, granulomatous formations have been related to heterogeneous drug distribution in tissues in particular in the context of tuberculosis where the drug was unable diffuse the necrotic core.^51^ For example, MALDI-MS was used to visualise the permeation of 279 compounds into the caseum of a tuberculosis lesion and demonstrated that caseum binding was directly related to a poor diffusion into the necrotic core which is key to resolve the infection.^67^ The large dataset further allowed the exploration of the physicochemical drivers of diffusion into the lesion core revealing high lipophilicity and poor solubility to stimulate binding to macromolecules while factors related to molecular shape (volume-to-surface ratio, number of aromatic rings) demonstrated an inverse correlation to free fraction. Other factors known to have impaired local drug perfusion includes pH as it impacts ionisation of the drug and thus molecule permeability across membranes^68^ – a phenomenon important in the context of ion trapping of drugs for intracellular pathogens.

Local inflammation in skin affects the size of the vessels and the bloodflow.^69^ Similarly in CL-affected skin, Wijnant *et al.* demonstrated an increased concentration of liposomal amphotericin B presumably due to an increased vascular permeability and increased macrophage influx.^19^ Further investigation of these observations compared a number of inflammation biomarkers between *L. major* infection characterised by a rapid onset and fulminant progressive infection and the more slowly progressing *L. mexicana* infection. A single dose of intravenous AmBisome led to increased amounts of AmB in infected skin, with twice as much AmB in the papule of *L. major* compared to the *L. mexicana*- infected skin.^19^ Localised CL pathology also showed deterioration of the skin barrier apparent by an increase in skin permeation of lipophilic (ibuprofen, log *D* = 4.0 (ref. 70)) and hydrophilic (caffeine, log *D* = −0.05 (ref. 71)) drugs topically applied to *L. major* infected compared to uninfected mouse skin.^18^

Relating immunopathology factors to clinical studies, the anti-inflammatory pentoxifylline shows a synergistic effect when co-administered with antimonial therapy in mucocutaneous patients in Brazil^72^ – this enhanced effect was not observed in CL patients.^73,74^ The distinct cytokine and macrophage population patterns between the different pathologies could explain the observed clinical outcomes but more research is required. Clinical studies equally revealed different cure rates for miltefosine administered orally in patients with mild (limited to nasal skin and mucosal tissue – 83% cure rate) and extended mucosal leishmaniasis (with involvement of palate, pharynx – 58% cure rate) caused by *L. braziliensis.* Clinical trials for CL often show differences in protocol and design, whereas these trials were conducted using similar methodologies involving the same causative species suggesting a causative relationship between the drug effectiveness discrepancies in different forms of CL and pathology potentially due to differences in drug distribution.^75,76^

However, measuring drug exposure in the dermis is challenging and often plasma concentrations are used as a surrogate of free drug concentrations in the dermis assuming a homogeneous and timely distribution to the active site. However, research investigating the drug distribution of anti-infective agents to various tissues including the skin exemplifies that this is not always the case.^77–79^ Hence, it important to stress the use of multiple techniques to understand the PK–PD relation of chemical series *in vitro* and *in vivo* using CL relevant models and how they correlate with antileishmanial effects (rate-of-kill) with the aim to accurately predict PK in man and maximise successful drug development.

### Drug development and formulation

**Fig. 3:**
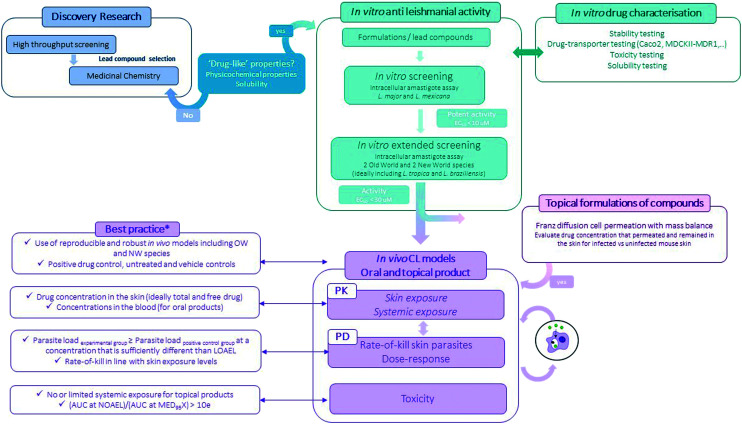
Flow-chart of the experimental preclinical drug discovery pathway for CL (*for more information see ref. 10).

Preclinical pathways for neglected diseases^8^ and specifically for CL have been mapped^10^ mainly focussing on medicinal chemistry. Even so, the attrition-rate of drugs in the development stage remains high. The importance of including formulation development (the process during which the API is combined with various chemical substances, including also excipients, to form the final medicinal product) is important to consider at this preclinical stage as it provides a critical link between the pharmacology, the pharmacokinetics and toxicology performance of the drug (Fig. 3).^80,81^ Excipients and formulations can influence the particle size, physical form, solubility and stability of the API thus impacting drug uptake and/or permeability across biological barriers and eventually efficacy and toxicity of the overall product.^80^

The earliest reports of topical treatment of tegumentary leishmaniasis date back to the 1920s describing the use of the trivalent antimony tartar emetic^82,83^ and the pentavalent stibosan. Other harsh chemicals were applied to the skin to combat the parasite including ointments consisting of sulphuric acid and charcoal (9 : 1). El-on (1984) however, was the first to evaluate a combination of antileishmanial compounds and excipients in a more conventional paraffin base for topical treatment of experimental CL.^84^ Application of Sb^v^ and Sb^III^ topically to the CL lesions resulted in a mean lesion size that was either similar (Sb^V^) or twice the size (Sb^III^) of the lesion of the untreated controls. In contrast, the application of 15% paromomycin and 12% methylbenzethonium chloride in a paraffin base onto the ulcer twice a day cured the *L. major* infection – results that spurred on clinical trials and resulted in the paromomycin containing ointment (Leshcutan®, Teva Pharmaceuticals) solely available in Israel. Other attempts were undertaken for example to reformulate already marketed antileishmanial drugs to modify the administration route from systemic into topical applications with limited success. The main reason is most likely the physicochemical nature of the molecules demonstrating high molecular weights and/or either a predominantly hydrophilic or hydrophobic nature – properties known not to facilitate skin permeability across the stratum corneum.^18^ A relatively simple experimental assay using Franz diffusion cells^85^ can help evaluate drug permeability across a membrane of interest. Extraction of the skin as a whole or distinct layers allows the evaluation of drug disposition profiles – a methodology frequently used to compare drug distribution profiles of distinct formulations.^33,86^

Besides the drugs highlighted above, topical formulations incorporating natural extracts have also been explored – some displaying promising results in *in vivo* CL models.^87–89^ It is important to note that commonly encountered problems such as (i) batch-to-batch variability of the extract, which may contribute to formulation and instability issues, (ii) availability of the plant material in sufficient quantities, and (iii) difficulties to identify the antileishmanial active molecule(s) compromise translatability and downstream development processes.

In contrast to conventional formulations, whereby a drug or novel antileishmanial compound is incorporated into a vehicle, encapsulating the drugs into nanoparticles modifies its physico-chemical properties. This strategy is especially promising for low-soluble compounds with poor absorption capacity as it can (i) modify PK properties of the drug, for example by sustaining release, (ii) enhance stability of the drug by offering protection against physical, chemical and enzymatic degradation, (iii) reduce toxicity^90,91^ and (iv) target infected cells and tissues. AmBisome is the only liposomal formulation available to treat leishmaniasis and is superior to other formulations of amphotericin B because of the significant reduction in toxicity. Many other nanoparticle formulations for both topical and systemic administration, have been tested against CL (for a comprehensive list see ref. 91) but none are currently in development. In fact, to date there are no FDA-approved topical nanoparticle formulations to treat skin diseases and clinical trials are equally lacking. Some of the main challenges that hinder development of nanoparticle formulations include toxicity, stability, cost and scaling-up for bigger batch preparation.

### New techniques to progress treatments for CL

Advances in technology are allowing in-depth analysis of cellular and micro-environmental changes before and after drug treatment. General research trends now include the evaluation of patient or animal samples using -omic, single cell, digital spatial profiling or MALDI-MS techniques followed by large data analysis and *in silico* modelling for hit identification which are subsequently verified using disease relevant *in vitro* or *in vivo* models. Some opportunities and techniques are described below.

#### Advanced *in vitro* models

The current *in vitro* models used to evaluate antileishmanial drug efficacy consist only of macrophages and intracellular amastigotes seeded on a 2D support. Modifications to investigate the impact of rate of flow of culture media and different scaffold support for macrophages on drug efficacy showed shifts in EC_50/90_ values for amphotericin B and miltefosine in cultures under medium flow.^52,92^ Further changes such as the addition of a second medium reservoir introducing fresh medium into the assay design, similar to the hollow fibre infection model, would enable the opportunity to test continuous *versus* intermittent drug exposure.^93^ Recent investigations demonstrate how the hollow fibre infection model can be used to reproduce pharmacokinetic profiles of clinically relevant anti-mycobacterial drug combinations, simulating drug levels at the target lung tissue.^94^ The possibility to mimic distinct micro-environments (such as oxygenation, protein binding, pH) or diverse drug regimens and subsequently tease apart the impact of these elements on the rate-killing of each drug as such or their combination remains difficult to conduct *in vivo* and makes this a powerful tool in drug discovery. Rate of kill studies would be improved by use of *Leishmania* parasites transfected with reporter genes that can indicate both *in vitro* and *in vivo* division rates, like the TIMER gene system used for *Salmonella* which showed different bacteria cell division rates in different tissues correlated with different antibiotic activities.^95^ The usage of the elegant biosensor *Leishmania* model that has a GFP reporter gene integrated within the 18S rDNA allows monitoring of the expression of 18S rRNA to test drug efficacy within quiescent populations.^96^

#### Digital spatial profiling (DSP)

DSP is a recently developed technique that allows multiplex and spatial detection and quantification of proteins or RNAs on formalin-fixed, paraffin-embedded samples. The approach relies on the multiplex regional readout of the target proteins or RNAs using oligonucleotide tags that are linked to antibodies or RNA probes through a photocleavable linker that is cleaved upon ultraviolet light exposure releasing the oligonucleotides in a spatial pattern across a region-of-interest consistent with the target location on the tissue section. This technology was successfully applied to CL patient samples from Sri Lanka before and after intra-lesional antimonial (SSG) treatment.^97^ Interestingly it validates previous findings of a drug-immune synergy whereby the early rounds of SSG injections reduce parasite burden alongside re-engagement of T cell effector function essential for parasite clearance and disease resolution. This opens opportunities to target pathways that accelerate micro environmental changes and minimise drug doses or treatment duration. Another application of technology could be the comparison for example of macular and polymorph forms of PKDL to identify key differences in pathology and disease driving pathways with the aim to develop drugs.

#### Matrix-assisted laser desorption/ionization mass spectrometry (MALDI-MSI)

Another technique to explore further is MALDI-MSI, which emerged as a label-free technology that can simultaneously map various biomolecules in cells and tissues with high sensitivity, specificity and relative quantitative abilities. Recent advances in different fields - sample preparation, instrumentation, quantification and large dataset profiling have led to a more frequent use of MALDI-MSI. A typical sample preparation involves the mounting of a cryosectioned slice of tissue onto a sample plate, which is coated with a suitable matrix that extracts analytes from the tissue of interest and co-crystalizes.^98^

Being able to discriminate samples based upon their chemical nature, MALDI-MSI has been used to detect differentially expressed peptides and low molecular proteins (2 to 20 kDa) in the liver upon infection with *L. infantum* in mice – this with the objective to evaluate pathophysiological changes and identify biomolecules that could serve as biomarker for diagnostic purposes.^99^ Interestingly, MALDI-MSI was able to generate quantitative skin distribution profiles for experimental psoriasis drug compounds from skin sections and could also distinguish drug permeation differences between different test formulations.^100^ This technique regardless of the laborious optimisation, would be a great addition to the arsenal of tools to study skin pharmacokinetics.

#### Microdialysis (MD)

Microdialysis (MD) is a minimally invasive technique for sampling free drug in the extracellular fluid within tissues.^101^ When inserted in the dermis, the probe, essentially a thin tubular semi-permeable membrane is slowly (0.5–10 μl per minute) perfused with a physiological solution. Only molecules smaller than the pore cut-off can diffuse from the tissue into the dialysate and with a slow perfusion rate, only small volumes of dialysate are collected for bioanalysis requiring sensitive analytical detectors. This technique was successfully applied to investigate the concentrations of DNDI-0690 in the dermal layers of the skin in an experimental CL model. The experiment aimed to answer three questions: (i) does DNDI-0690 distribute to the skin, (ii) which administration route, topical or oral, is most suitable and, (iii) does pathology impact skin drug distribution.^32^ Skin microdialysis and Franz diffusion cell studies revealed that DNDI-0690 permeated poorly into healthy and diseased skin upon topical application of a saturated solution. An oral dose of 50 mg kg^−1^ instead lead to rapid distribution of protein unbound DNDI-0690 from the blood into the infected dermis as indicated by a ratio of the area under the curve (0 to 6 hours) of free DNDI-0690 in the skin to that in the blood greater than 80%. Bioluminescence imaging also indicated that two oral doses (50 mg kg^−1^) led to a 2 log fold reduction of the *L. mexicana* parasite load whereas 6 doses were needed for a similar reduction in *L. major*.

Proof-of-concept studies in post-kala azar dermal leishmaniasis patients showed that miltefosine concentrations in the skin could successfully be measured – this technique offers the opportunity to investigate the relation between drug exposure in the plasma and the dermis and would allow to optimise drug regimens based on PK parameters rather than toxicity (Wijnant, Moulik, Van Bocxlaer, Chatterjee, Das, de la Flor, Chatterjee and Croft, unpublished). In addition, open-perfusion skin microdialysis whereby the probe membrane is replaced by a steel mesh featuring macroscopic openings, would have allowed quantification of high molecular weight molecules in conjunction to free drug fractions in the dermis.^102^ This is particularly useful to measure cytokine profiles in reaction to treatment which could help evaluate immunomodulators and therapeutic antibodies^103^ in parallel to measuring disease biomarkers.^104^

A major downside to this technique is the extensive optimisation that is required. To be able to relate dialysate concentrations back to absolute drug amounts, the *in vitro* relative recovery is calculated in an *in vitro* experiment by measuring the relative loss of a compound from the perfusate. Further it is important to establish the depth of the probe insertion to ensure that drug sampling is conducted in the actual drug target compartment.^105^

## Conclusion

Cutaneous leishmaniasis does not cause fatalities but infection leads to significant morbidity related to disfiguration and social stigma. A selection of drugs, mainly repurposed, are available but due to toxicity, cost and variable efficacy, patients delay seeking treatment and allow the infection to progress increasing the risk of parasite spread, expansion of the lesion size and number, aggravation to the ulcer stage and contribution to transmission. An effective and safe treatment is needed to change the treatment seeking behaviour and avoid scar formation.

Currently however, the development of novel treatments is mainly focussed on VL. Two new chemical series, the benzoxaboroles and nitroimidazoles with DNDI-6148 and DNDI-0690 as lead compounds respectively, showed excellent activity in experimental models of both VL^106^ and CL^32,50^ and have now progressed into clinical phase 1 trials. In the meanwhile, back-up series are being secured to address the attrition rate inherent to R&D activities and new chemical series are being explored *via* compound library screenings enabled through partnerships with pharma and biotech companies. For example, a screening programme with Pfizer revealed aminopyrazole compounds with potent activity against VL^107^ and CL^50^ strains both *in vitro* and *in vivo* and a partnership with GSK resulted in the identification of the pyrazolopyrimidine scaffold as another potential chemical series with antileishmanial activity (lead: DDD853651/GSK3186899).^108^ At the same time research at Novartis identified a novel series of compounds of anitleishmanial compounds, with novel selective proteasome inhibition, of which a derivative LXE408 is now in Phase 1 trials.^109^

With these potent VL drugs in the pipeline, it is now important to evaluate the efficacy of these drugs against CL. This chapter describes and exemplifies some of the core strengths and shortcomings of the commonly used experimental models for CL but most importantly, it highlights how each of the available tools together and in combination with in-depth analysis allows a PK/PD integrated drug discovery and development approach essential to guarantee clinical success.

## Conflicts of interest

There is no conflict of interest to declare.
